# Institutional Trend in Device Selection for Transcatheter PDA Closure in Premature Infants

**DOI:** 10.1007/s00246-022-02903-2

**Published:** 2022-04-16

**Authors:** Peter Guyon, Nicole Duster, Anup Katheria, Caitlyn Heyden, Danica Griffin, Ronald Steinbergs, Andres Moreno Rojas, Kanishka Ratnayaka, Howaida G. El-Said

**Affiliations:** 1 Division of Pediatric Cardiology, Rady Children’s Hospital | UC San Diego School of Medicine, 3020 Children’s Way MC #5004, San Diego, CA 92123 USA; 2 Department of Pediatrics, Rady Children’s Hospital | UC San Diego School of Medicine, San Diego, CA USA; 3 Department of Neonatology, Sharp Mary Birch Hospital for Women and Newborns, San Diego, CA USA

**Keywords:** Prematurity, Patent ductus arteriosus, Transcatheter

## Abstract

We report our experience with transcatheter patent ductus arteriosus (PDA) closure in premature infants and compare patients grouped by the device used for closure: the Microvascular Plug, “MVP” (Medtronic, Minneapolis, MN); Micro Plug Set, “Micro Plug” (KA Medical, Minneapolis, MN); and Amplatzer Piccolo Occluder, “Piccolo” (Abbot, Santa Clara, CA). We also report trends in device selection over time. Studies examining outcomes according to device selection for PDA closure in premature infants are lacking. We performed a retrospective review of all percutaneous PDA closures in premature infants at a single center (June 2018–May 2021). Patients were grouped by initial device selected for PDA closure (intention to treat). Institutional Review Board approval was obtained. 58 premature infants [MVP (*n* = 25), Micro Plug (*n* = 25), and Piccolo (*n* = 8)] underwent successful transcatheter PDA closure (mean gestational age 27 weeks 2 days; mean weight at procedure 1.4 kg; mean age at procedure 28 days). Pre-procedural demographics, procedural data, and follow-up data were similar between groups. There were no significant procedural adverse events. Three devices (2 MVP, 0 Micro Plug, 1 Piccolo *p* = 0.27) embolized after the procedure. One other device was removed for concern for aortic obstruction. Device selection evolved with a clear trend toward the Micro Plug device over time. Demographic, procedural, and follow-up data were similar between the MVP, Micro Plug, and Piccolo groups. The Micro Plug did not require exchange for suboptimal fitting or embolize and became our preferred device in most cases.

**Supplementary Information:**

The online version contains supplementary material available at 10.1007/s00246-022-02903-2.

## Introduction

Patent ductus arteriosus (PDA) is common in premature infants, with an estimated 20%–60% incidence and an inverse relationship to birth weight [1]. Hemodynamically significant PDA causes a left-to-right shunt with multiple potential physiologic effects, including pulmonary over-circulation with left heart overload or heart failure, as well as shock from low cardiac output. Additionally, persistent PDA is associated with bronchopulmonary dysplasia, sepsis, necrotizing enterocolitis, infective endocarditis, and pulmonary hypertension [2]. In one study, the adjusted hazard for death in preterm neonates with PDA was eightfold higher than those with a closed ductus [3].

Treatment of PDA begins with medical therapy using cyclooxygenase inhibitors, including indomethacin and ibuprofen, as well as acetaminophen [4]. Surgical ligation was previously the definitive management for hemodynamically significant PDA that were refractory to medical therapy, although currently the indications for surgery are controversial given the associated comorbidities [5].

Advances in technique and the proliferation of available devices have led to success with transcatheter management of PDA even in very small premature infants, and transcatheter closure is an increasingly accepted practice [4, 6–16]. In the last several years, success has been reported using the Medtronic Micro Vascular Plug “MVP” (Medtronic, Minneapolis, MN) [9, 11], the purpose-built Amplatzer Piccolo Occluder “Piccolo” (Abbot, Santa Clara, CA) [12–15], and the Micro Plug Set “Micro Plug” (KA Medical, Minneapolis, MN) [17]. No studies to date have compared the safety or clinical merits of these different devices. This retrospective review reports the experience with transcatheter PDA occlusion at our institution using these three devices. We compare the safety and efficacy between device types and demonstrate an evolution in device selection over time, with the Micro Plug device emerging as the preferred device for our institution. This paper also serves as an additional report of successful use of the Micro Plug device in a larger number of patients than the original report (*n* = 8) by our group [17].

## Materials and Methods

We performed a retrospective review of all ex-premature infants who underwent transcatheter closure of PDA in the cardiac catheterization lab at a single center beginning in June 2018 and ending May 2021. Three experienced operators contributed all their respective cases for analysis. Institutional Review Board approval (University of California, San Diego) was obtained with a waiver of consent. Patient data including demographics, cardiac catheterization reports, angiography, echocardiogram reports, and clinical notes were analyzed. The Piccolo device received FDA approval specifically for closure of PDA in small infants in January 2019. The MVP and Micro Plug devices received FDA approval in December 2013 and May 2019, respectively, for other indications. Our operators used the latter two devices “off-label” for premature PDA closure based on the clinical situation (including patient size and ductal anatomy). During consent, patient families were counseled about the potential use of different device types based on the in-procedure findings.

### Statistical Methods

The cohort was separated into three groups for comparison based on the first device selected for closure of the PDA (intention to treat). The three groups were compared using ANOVA (analysis of variance) for numerical data and the Kruskal–Wallis Rank Sum Test for categorical data. Statistical analysis was performed using R version 4.0.5 (R Foundation for Statistical Computing, Vienna, Austria). Cases in which the first device selected for closure was ultimately not used and an alternate device was needed were tracked and reported. Evolution of device selection over time was tracked by noting the number of devices initially selected for closure during each semester of the study period. (The initial “semester” was one month and the final “semester” was 5 months, as data collection initiated in June 2018 and finished in May 2021.)

## Results

A total of 58 ex-premature infants underwent transcatheter PDA closure over a three-year time interval (06/2018 and 05/2021). Overall, the mean gestational age was 27 weeks 2 days, mean birth weight was 1.0 kg, mean weight at procedure was 1.4 kg, and mean age at procedure was 28 days. Initial devices selected for closure were MVP *n* = 25; Micro Plug *n*  = 25; and Piccolo *n* = 8. There was no statistically significant difference between the three groups based on the initial device selected for closure in terms of pre-procedural demographics (gestational age, birth weight, sex), procedural factors (age and weight at procedure, level of respiratory support, dimensions of PDA by angiography, fluoroscopy time, radiation), and follow-up (time to extubation, estimated peak pressure gradient in the aorta and left pulmonary artery as measured by Doppler echocardiogram) (Table 1). The same basic procedural algorithm was followed regardless of device selected, including use of angiograms and use of echocardiography.

**Table 1:** Demographic, procedural, and follow-up data

	MVP	Micro plug	Piccolo	*p* value
*n* (58)	25	25	8	
Demographic data				
Mean gestational age	27w6d	27w0d	26w5d	***0.46***
Mean birth weight (kg)	1.1	0.99	0.91	***0.53***
Sex (% female)	56%	60%	50%	***0.88***
Procedure data				
Mean weight at procedure (kg)	1.51	1.40	1.31	***0.78***
Mean age at procedure (days)	26	30	28	***0.72***
Respiratory support				***0.72***
Conventional vent	11	8	4	
Oscillator	5	3	1	
CPAP	8	7	2	
Nasal Cannula	1	2	1	
None	0	3	0	
Not reported	0	2	0	
Mean PDA size aortic end (mm)	3.9	3.4	3.2	***0.18***
Mean PDA size minimum (mm)	2.5	2.3	2.2	***0.68***
Mean PDA length (mm)	10.2	9.4	8.2	***0.19***
Fluoro time (min)	7.7	9.2	10.2	***0.16***
Radiation DAP (cGy*cm2)	42.9	40.2	38.3	***0.96***
Follow-up data				
Mean time to extubation (days)	10	6	12	***0.37***
Peak gradient (Doppler descending aorta, mmHg)	6.4	5.5	4.6	***0.35***
Peak gradient (Doppler left pulmonary artery, mmHg)	5.5	7.1	5.2	***0.39***

**Fig. 1:**
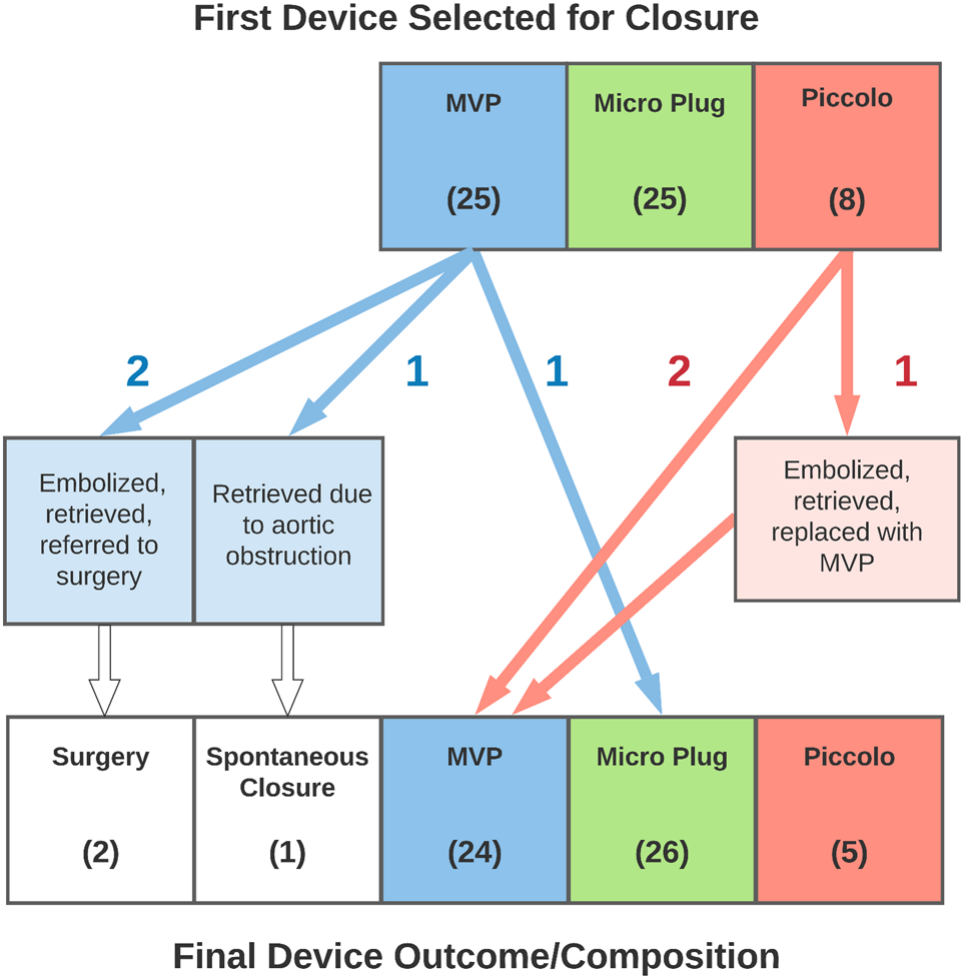
Complete Cohort information. Graphical representation of device outcomes beyond the initial intention-to-treat analysis. Two MPV devices embolized and were retrieved; those patients were referred to surgery. One MVP device was electively retrieved due to a persistent increased Doppler flow velocity in the descending aorta and that patient’s PDA then spontaneously closed. One MVP device was selected and deployed based on initial angiography; however, due to suboptimal positioning it was removed, and a Micro Plug device was deployed and released instead. All Micro Plug devices were successfully released; none embolized. Two Piccolo devices were selected and deployed based on initial angiography; however, due to suboptimal positioning they were removed, and MVP devices were deployed and released instead. One Piccolo device embolized and then an MVP device was successfully placed during the transcatheter retrieval procedure

A PDA closure device was successfully deployed and released in all 58 cases. There were no major procedural adverse events. There were four total major post-procedural adverse events (6.9% incidence). Three devices embolized in the immediate peri-operative period (2 MVP, 0 Micro Plug, 1 Piccolo *p* = 0.27.) One device embolized in the procedure room while moving the patient from the procedure table to the transporter isolette. The other two embolized to the left pulmonary artery after transport to the neonatal intensive care unit (NICU) and were discovered by the initial post-procedure X-ray performed within 90 min of the procedure. All three embolized devices were successfully retrieved via transcatheter approach in the catheterization lab on the same day as the initial procedure. Two of the patients were referred for surgical ligation; the third patient had successful transcatheter closure of the PDA using another device type during the retrieval procedure. The fourth adverse event consisted of an elective removal of an MVP device by transcatheter snare two days after the procedure. The device was removed based on increased echocardiographic Doppler gradient in the descending aorta at 48-h post-procedure (41 mmHg peak gradient) and a clinical upper-to-lower extremity blood pressure gradient of 20 mmHg. After device removal, the ductus spontaneously closed, so no additional procedure was needed (Fig. 1).

There were two mortalities during the study. One was an infant born at 31 weeks 5 days gestational age weighing 2.4 kg diagnosed with Beckwith Weidman Syndrome, who also had a ruptured omphalocele, recurrent episodes of sepsis, renal failure, and a large PDA. The patient underwent transcatheter PDA closure on day of life 38, the device embolized was retrieved by percutaneous approach without incident, and the patient was then referred to surgery based on the very large size of the ductus. The patient died 10 days after surgical ligation when parents requested life support be withdrawn rather than proceeding with dialysis and more interventions. The other mortality occurred in an infant born at 25 weeks 6 days gestational age weighing 900 g with a grade III intraventricular hemorrhage which was diagnosed day of life 4 (nine days prior to the catheterization). The patient underwent an uneventful catheterization (heparin was not given per the institutional protocol) and the patient was transported back to the NICU in stable condition. Two days after PDA closure, the patient was compassionately extubated according to parents’ wishes for comfort care due to ongoing respiratory failure, seizures, and declining neurologic status related to the intraventricular hemorrhage.

Three events were considered clinically notable but did not rise to the level of adverse events: three patients were seen to have new mild tricuspid regurgitation on the echocardiogram in the immediate post-procedure period (1 MVP, 1 Micro Plug, 1 Piccolo *p* = 0.62). However, in all cases the degree of tricuspid regurgitation had returned to trivial on follow-up echocardiograms performed more than 2-month post-procedure.

The initial device selected for deployment based on angiography was not always the final device which was deployed. In our cohort, the first device selected was deployed and released 83% of the time (48/58); in the other 17% of cases, the initial device was removed before it was released and replaced by another size of the same device type or replaced by a different device type. One out of 25 MVP devices was deployed but not released (the MVP device caused some aortic obstruction on echocardiography) and then was replaced with a Micro Plug device. Two out of 8 Piccolo devices were deployed but not released (one due to partial aortic obstruction, the other due to partial aortic obstruction, followed by left pulmonary artery obstruction after repositioning by echocardiogram) and then were replaced with MVP devices. One Piccolo device which embolized was replaced with an MVP device during the retrieval procedure. Notably, embolization occurred after closure with the MVP (2) and Piccolo (1) devices, but not with the Micro Plug device (although this finding did not reach statistical significance). Additionally, it is notable that some of the Micro Plug devices were not deployed and were replaced with Micro Plug devices of a different size, but none were replaced by different device types (Fig. 1).

**Fig. 2:**
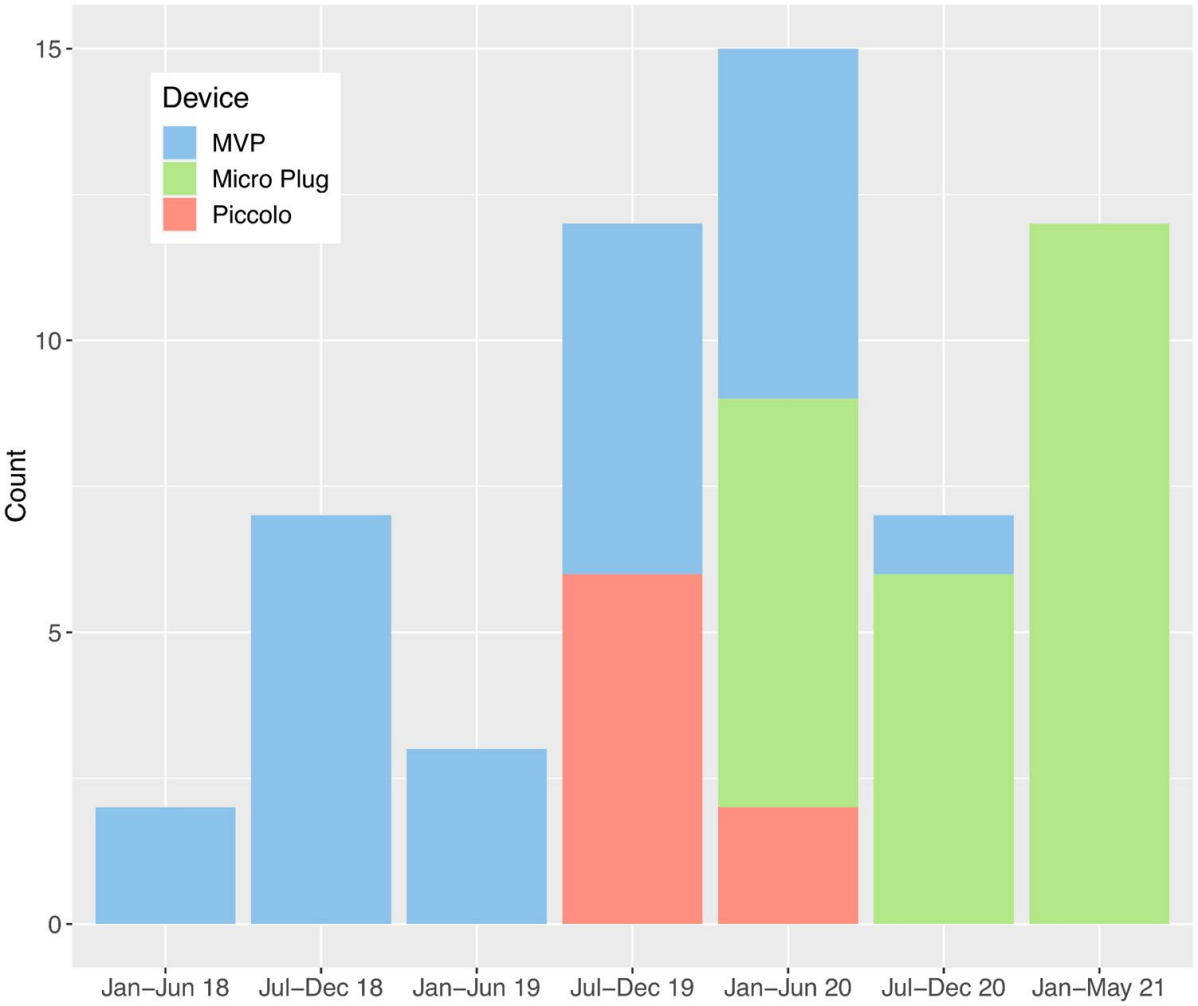
Device selection over time. Stacked bar which displays the initial device selected for premature PDA closure by semester during the study period. In the early experience, the MVP was favored. In the more recent experience, there is a preference toward selection of the Micro Plug device.

The initial device selected for PDA closure changed over time: the early experience consisted of exclusive use of the MVP device, with subsequent introduction of the Piccolo and finally the Micro Plug device. Beginning in October 2020 to the end of the study period, the Micro Plug device was selected exclusively (15 consecutive cases) for use with this procedure at our institution (Fig. 2).

Mean duration of follow-up time for the entire cohort was 159 days (range 42–660). At the time of most recent follow-up, the groups were similar in terms of outcomes: there was no significant obstruction to flow in the descending aorta or the left pulmonary artery overall and no difference between the groups when comparing mean Doppler measurements of peak gradient in the descending aorta or the left pulmonary artery (Table 1).

## Discussion

### Safety and Efficacy

Transcatheter closure of PDA was performed successfully in 58 premature patients using any of three commercially available devices with no major procedural adverse events. Using criteria from a recent meta-analysis of transcatheter PDA closure in infants weighing 1.5 kg or less [18] (Table 1), there were four major adverse events noted in the post-procedural period. There was one elective device removal for partial obstruction to flow in the descending aorta and three other cases of device embolization requiring an additional procedure for retrieval. The 6.9% incidence of major adverse events is in line with the 8% incidence quoted in the above-cited meta-analysis, which included closures performed using the Piccolo device, the MVP, the Amplatzer Vascular Plug II (AVP II; *Abbott*, Santa Clara, CA), and various coils [18]. Three cases of new, mild tricuspid regurgitation seen on the immediate post-procedural echocardiogram were not considered major or adverse events as they did not meet the criteria cited above, did not require any treatment or intervention beyond the normal for the group, and resolved by 2–3-month follow-up. None of the embolized devices required surgical retrieval and none of the patients experienced significant hemodynamic instability or long-term morbidity from the events. The two mortalities occurred in patients with additional medical comorbidities and were not related to the catheterization procedure.

Overall, these results demonstrate a favorable safety and efficacy profile for the procedure using any of the devices and are consistent with previous reports [9–11, 13–15, 17]. We note that in our cohort, embolization occurred after deploying and releasing the MVP device and the Piccolo device, but not the Micro Plug device. Because the number of adverse events is small it is difficult to draw strong conclusions from this finding, which did not reach statistical significance. The operators posit that the relative softness of the microcatheter during delivery, which creates less distortion of the anatomy, offers more precision when positioning the device, leading to a reduced incidence of embolization. Our group previously reported successful use of the Micro Plug device off-label for percutaneous closure of PDA in premature infants in a small cohort of 8 patients [17]. This report provides additional evidence of the safety and efficacy of using the device for this indication and shows that it compares favorably to the purpose-built Piccolo device and MVP device.

Although the cohort was analyzed using an intention-to-treat model, the first device selected for closure based of angiographic measurements was not always the one which was ultimately released. In our cohort, the first device selected was deployed and released 83% of the time (48/58); in the other 17% of cases, the initial device was removed before it was released and replaced with a different device type or another size of the same device type. This is consistent with anticipated difficulty in accurately measuring the size of a PDA, given the propensity to spasm and relax, potentially causing over- or underestimation of the size of the vessel. All three device types were replaced by a different size of the same device type before release. Notably, one MVP device was removed before release and replaced by a Micro Plug device; three Piccolo devices were removed before release and replaced by MVP devices; no Micro Plug devices needed to be removed and replaced with a different device type (although there were cases where the first Micro Plug device replaced another Micro Plug device of a different size). In the final analysis, 24 MVP, 26 Micro Plug, and 5 Piccolo devices were in place; 2 patients underwent surgery after embolization and one patient’s ductus closed spontaneously after embolization (Fig. 1).

### Trend Toward Preferred Use of Micro Plug Device

There was a clear institutional trend over time when selecting the initial device for PDA closure. At the beginning of the group’s experience with premature infant PDA closure and through the first half of 2019, the MVP device was selected exclusively. The second half of 2019 saw the introduction of the Piccolo device, and early 2020 saw the introduction of the Micro Plug device, after which all three devices were used for a time. The second half of 2020 and early 2021 saw almost exclusive selection of the Micro Plug device for use.

There are several reasons for the institutional trend toward preferred use of the Micro Plug. The short length of the Micro Plug device, like the Piccolo, provides protection against protrusion into the aorta or left pulmonary artery. In addition, the Micro Plug device, like the MVP, is delivered through a soft microcatheter rather than a relatively stiff delivery sheath. Our operators observed increased confidence delivering the device through a microcatheter, since its softness and trackability protected against distortion of the anatomy and resulting hemodynamic instability during device deployment when compared to the delivery sheath required by the Piccolo. Both the Micro Plug device and Piccolo offer an advantage of improved visibility under fluoroscopy during deployment and release when compared to the MVP device. One additional factor is cost, where the MVP and Micro Plug devices cost significantly less when compared to the Piccolo device. Comparing all these factors, the Micro Plug device best combined the strengths (short length, delivery through a microcatheter, less distortion of anatomy during deployment, adequate visibility on fluoroscopy, lower cost), making it the preferred device in most situations.

However, each device does offer potential strengths and weaknesses which should be considered when contemplating premature infant PDA closure. Other important factors when planning for a device closure in addition to those listed above include the available sizes of the device (diameter, as well as length) and the ability to see the device by echocardiography. For instance, a large-diameter PDA might dictate that an MVP device should clearly be used, as it offers the largest diameter size by far (maximum 9 mm compared to maximum 5 mm Piccolo and 6 mm Micro Plug). We offer a comparison of device attributes based on our single-center experience in Table 2. Additionally, since our center now has the most published experience using the Micro Plug device for premature PDA closure, we offer a guideline for choosing a Micro Plug size based off the minimum PDA diameter measured by angiogram (Table 3). This information may be valuable for other programs which perform transcatheter PDA closure in premature infants.

**Table 2:** Comparison of device attributes

	Echo visibility	X-Ray visibility	Trackability	Microcatheter deployment	Available lengths (unconstrained)	Available diameters	Cost
MVP	−	+	+++	Yes	12–16 mm	3 mm 5 mm 7 mm 9 mm	$
Micro Plug	++	++	+++	Yes	2.5 mm	3 mm 4 mm 5 mm 6 mm	$
Piccolo	++	+++	+	No	2, 4, 6 mm	3 mm 4 mm 5 mm	$$$

**Table 3:** Micro plug size guide

PDA minimum diameter (mm)	Micro plug device size
1–1.2	3
2–3.2	4
3–4.2	5
4–5.2	6

### Limitations and a Word of Caution

The authors note that since this study was a retrospective review at a single institution, it is subject to potential bias and confounding. Additionally, a robust statistical comparative analysis between the devices is hindered by the small sample size. As noted in the “Materials and Methods” section, the Piccolo device received FDA approval for PDA closure in premature infants, while the other devices have not been approved for this particular indication and are being used “off-label.” Operators must take this into account when selecting inventory and when counseling patient families.

## Conclusion

Closure of a PDA in 58 premature infants was successful using three commercially available devices, all with a favorable safety profile. Over time, our group has gravitated toward selecting the Micro Plug device in most cases because it is short, visible, trackable, delivered through a soft microcatheter, and relatively inexpensive. This comparison of three devices is based on a retrospective analysis with small numbers and thus should be interpreted with caution. 

## Supplementary Information

Below is the link to the electronic supplementary material.

Supplementary file1 (XLSX 18 kb)

Supplementary file2 (PDF 462 kb)

Supplementary file3 (PDF 17 kb)
