# Supplementary material for: Institutional Trend in Device Selection for Transcatheter PDA Closure in Premature Infants
Source: Pediatr Cardiol. 2022 Apr 16;43(8):1716–22. doi: 10.1007/s00246-022-02903-2 (PMC9587941; doi:10.1007/s00246-022-02903-2)
Supplement: Supplementary file 2 — Supplementary file2 (PDF 462 kb) [file 246_2022_2903_MOESM2_ESM.pdf]

KA MEDICAL

# US Customer Set-up and Agreement

## ENTITY INFORMATION

| | | | | | | | |
|---|---|---|---|---|---|---|---|
| Exact Legal Name of Entity: | | | | | | | |
| List any other Trade Names, DBA's: | | | | | | | |
| **Customer Address** | | | | **Delivery Address** ☐ Same as Customer Address | | | |
| Street Address | | | | Street Address | | | |
| Address Line 2/Department | | | | Address Line 2/Department | | | |
| City | State | Postal Code | Country | City | State | Postal Code | Country |
| **Invoice Address** ☐ Same as Customer Address | | | | | | | |
| Street Address | | | | Type of Business: ☐ Hospital ☐ Manufacturer ☐ Surgery Center ☐ Distributor ☐ Contractor ☐ Dealer ☐ Other (Explain):______ | | | |
| Address Line 2/Department | | | | | | | |
| City | State | Postal Code | Country | | | | |
| Federal Tax ID No. (if different from VAT No.) | | VAT No. | | | Local Currency Credit Limit Requested: | | |

## ENTITY REPRESENTATIVES INFORMATION

| | | | |
|---|---|---|---|
| **Primary Physician Contact Information** | | **Accounts Payable Contact Person** | |
| Name: | | Name: | |
| Phone: | Fax: | Phone: | Fax: |
| Email: | | Email: | |

## CUSTOMER AUTHORIZATIONS AND SIGNATURE

KA Medical, LLC's payment terms are Net 30 Days from date of invoice. All invoices are due on the due date. If any sections of this form are not completed, order processing, credit consideration and allocation may be adversely affected.

The entity representative's signature, on behalf of the debtor, attests financial responsibility, ability, and willingness to pay in accordance with the above standard payment terms. Applicant, and the officer or other representative signing on behalf of applicant, hereby certify that they have carefully reviewed this form and it is warranted to be true and complete.

Authorized Representatives Signature:________________ Date: __________

Print Name:________________ Title: __________

**Return completed form to info@ka-medical.com**

## FOR KA MEDICAL USE ONLY:

| | |
|---|---|
| Customer No. | Authorized Credit Limit: |

KA Medical Representatives Signature: ________________ Date: __________

Print Name: ________________ Title: __________

Add to customer master: Initials: ________ Date: __________



KA Medical, LLC
2890 Centre Pointe Drive
Roseville, MN 55113 USA

# US Order Instructions and Price List

## ORDER INSTRUCTIONS

To place an order or request a quote please

contact: Email: info@ka-medical.com

Phone: 651-262-2810

## PRICE LIST

| Order Number | Description | Price (each) |
|---|---|---|
| **90000** | **Micro Plug Set, 3mm Plug, 125cm Catheter** | **$1400.00** |
| **90001** | **Micro Plug Set, 4mm Plug, 125cm Catheter** | **$1400.00** |
| **90002** | **Micro Plug Set, 5mm Plug, 125cm Catheter** | **$1400.00** |
| **90003** | **Micro Plug Set, 6mm Plug, 125cm Catheter** | **$1400.00** |

**Note: Price does not include shipping and handling.**

**Approved for ordering, returned to customer: Init: _______________ Date: __________________________**



KA Medical, LLC
2890 Centre Pointe Drive
Roseville, MN 55113 USA
