# Supplementary material for: Institutional Trend in Device Selection for Transcatheter PDA Closure in Premature Infants
Source: Pediatr Cardiol. 2022 Apr 16;43(8):1716–22. doi: 10.1007/s00246-022-02903-2 (PMC9587941; doi:10.1007/s00246-022-02903-2)
Supplement: Supplementary file 3 — Supplementary file3 (PDF 17 kb) [file 246_2022_2903_MOESM3_ESM.pdf]

EXHIBIT A

PRODUCT DESCRIPTION AND PRICING

Model Number	Model Description	Unit of Measure	Customer Price
9-PDAP-03-02-L	AMPLATZER Piccolo 3-2mm	EA	$9,680.00
9-PDAP-03-04-L	AMPLATZER Piccolo 3-4mm	EA	$9,680.00
9-PDAP-03-06-L	AMPLATZER Piccolo 3-6mm	EA	$9,680.00
9-PDAP-04-02-L	AMPLATZER Piccolo 4-2mm	EA	$9,680.00
9-PDAP-04-04-L	AMPLATZER Piccolo 4-4mm	EA	$9,680.00
9-PDAP-04-06-L	AMPLATZER Piccolo 4-6mm	EA	$9,680.00
9-PDAP-05-02-L	AMPLATZER Piccolo 5-2mm	EA	$9,680.00
9-PDAP-05-04-L	AMPLATZER Piccolo 5-4mm	EA	$9,680.00
9-PDAP-05-06-L	AMPLATZER Piccolo 5-6mm	EA	$9,680.00
